# Physical activity levels and quality of life in osteoporosis patients

**DOI:** 10.3389/fspor.2026.1766982

**Published:** 2026-06-23

**Authors:** Sarit Bregman, Lynn Smith

**Affiliations:** Department of Sport and Movement Studies, Faculty of Health Science, University of Johannesburg, Johannesburg, South Africa

**Keywords:** biokinetics, GPAQ, OPAQ, osteoporosis, physical activity, quality of life

## Abstract

**Background:**

Physical activity has been shown to support the maintenance of bone mineral density and reduce bone loss, particularly in older adults and individuals with osteoporosis, while also improving functional capacity and quality of life.

**Objective:**

This study aimed to determine physical activity levels and quality of life among adults with osteoporosis aged ≥35 years living in Johannesburg, South Africa. It was hypothesised that higher physical activity levels would be associated with improved quality-of-life outcomes.

**Methods:**

A quantitative, cross-sectional study was conducted using the Global Physical Activity Questionnaire (GPAQ) and the Osteoporosis Assessment Questionnaire Version 2 (OPAQ 2.0). A total of 209 participants (mean age: 64 years) completed the combined questionnaire. Descriptive statistics, chi-square tests, independent t tests, and correlation analyses were performed.

**Results:**

Most participants met physical activity guidelines through work-, transport-, or leisure-related activity, although vigorous-intensity activity was limited. Participants using private healthcare reported higher overall quality of life scores (73%) than those using public healthcare services (50%). The physical-functioning domain was rated highest (76%), followed by emotional well-being (58%) and social interaction (56%). Increased physical activity levels were associated with better quality of life outcomes.

**Conclusion:**

Although many participants demonstrated adequate physical activity levels, sedentary behaviour remained prevalent. Quality of life was most negatively impacted in the emotional and social domains. These findings highlight the potential importance of physical activity, particularly structured load-bearing exercise, in osteoporosis management and overall well-being. This study contributes rare South African data on osteoporosis, physical activity behaviour, and the potential role of biokinetics within both private and public healthcare settings.

## 1 Introduction

Osteoporosis is a systemic skeletal disorder characterised by reduced bone mass and microarchitectural deterioration, leading to increased bone fragility and heightened fracture risk (1, 2). Globally, an estimated 37 million fragility fractures occur annually among adults aged ≥55 years, and lifetime risk projections indicate that one in three women and one in five men over 50 will experience an osteoporotic fracture, contributing significantly to disability and diminished quality of life (1, 3, 4). With population ageing, the absolute number of fractures and years lived with disability is rising globally, reflecting a growing public health burden (3). Although substantial international data describing osteoporosis prevalence and fracture incidence are available, epidemiological evidence from African countries, including South Africa, remains comparatively limited. Nevertheless, recent South African estimates suggest a substantial and increasing burden of osteoporosis-related fractures, with annual hip fractures projected to rise from approximately 11,000 in 2020 to 26,400 by 2050 (5).

Studies in sub Saharan Africa reveal that osteoporosis prevalence and fragility fracture incidence are not negligible, though high-quality local data are scarce (6). Vertebral fractures, among the most common and disabling osteoporotic fractures, are also frequently underdiagnosed, underscoring an unmet need for improved detection and management (1).

Osteoporotic fractures can severely impair physical functioning, mobility, and activities of daily living, often resulting in chronic pain, loss of independence, fear of falling, and social withdrawal. These consequences may discourage engagement in physical activity, thereby accelerating declines in bone mineral density (BMD), muscle strength, balance, and overall function, increasing the risk of subsequent falls and fractures. Physical activity interventions, particularly weight-bearing, resistance, and impact exercises, have been shown to improve bone health by enhancing BMD, muscle strength, coordination, and balance, and may contribute to improved quality of life in older adults (7, 8). Importantly, in older adults and clinical populations, physical activity may play a greater role in maintaining bone mass and reducing bone loss rather than substantially increasing BMD. Despite evidence supporting the benefits of physical activity in osteoporosis, few studies have examined physical activity patterns or QoL outcomes specifically among adults with osteoporosis in South Africa, where healthcare access inequalities may influence diagnosis, management, and rehabilitation opportunities (9).

Given the substantial disparities between public and private healthcare systems in South Africa, differences in access to rehabilitation services, health promotion initiatives, and osteoporosis management may influence physical activity participation and quality-of-life outcomes among affected individuals. This study aimed to determine physical activity levels and quality of life among adults with medically diagnosed osteoporosis living in Johannesburg, South Africa, to inform targeted interventions and improve health outcomes. It was hypothesised that higher physical activity participation would be associated with improved quality-of-life outcomes.

## 2 Materials and methods

### 2.1 Study design and participants

A quantitative, descriptive cross-sectional design was used. Purposive and snowball sampling methods recruited 209 adults (≥35 years) with medically diagnosed osteoporosis living in Johannesburg. Adults aged ≥35 years were included to accommodate individuals with early-onset and secondary osteoporosis, which may occur before older adulthood due to medical, endocrine, autoimmune, medication-related, or lifestyle-associated factors. Although osteoporosis is more prevalent in older populations, the inclusion of younger adults allowed for broader representation of clinically diagnosed cases. Osteoporosis diagnosis was based on prior clinical diagnosis reported by participants; however, subtype classification (e.g., primary vs. secondary osteoporosis) was not consistently available, and both forms may therefore have been represented within the sample. Participants were recruited from both public and private healthcare settings, including a tertiary hospital osteoporosis clinic, private practices, and online platforms, to improve sample diversity and capture a broader representation of adults with osteoporosis across different healthcare contexts within Johannesburg. Although both males and females were eligible for participation, the final sample was predominantly female, and sex-stratified analyses were not performed. The online questionnaire link was distributed via social media platforms and shared with endocrinologists, physiotherapists, and biokineticists, who further disseminated it to eligible patients.

### 2.2 Ethical considerations

Prior to data collection, ethical approval was obtained from the relevant institution's research ethics committee (REC-1053-2023). Clearance was also granted by the National Health Research Authority for access to collect data at public healthcare facilities (NHRD-GP_202105_063). Additional ethical approval was obtained from the relevant hospital division and its affiliated university's medical ethics committee (M220602 MED22-05-068). Participant responses were anonymised prior to analysis and stored on password-protected institutional devices accessible only to the research team. All data handling procedures complied with institutional ethical requirements and applicable data protection principles.

### 2.3 Data collection

No identifying information was collected, and all questionnaires were completed anonymously. Two validated instruments were combined into a single survey. The first component was the Global Physical Activity Questionnaire (GPAQ) (10), which assesses physical activity across three domains, namely; work-related activity, travel-related activity, and leisure-time activity. It evaluates both moderate- and vigorous-intensity activity within each domain. GPAQ responses were scored according to World Health Organization guidelines. Moderate-intensity activities were assigned 4 metabolic equivalents (METs) and vigorous-intensity activities were assigned 8 METs. Total physical activity was calculated as MET-minutes per week. Standard GPAQ truncation procedures and validity checks were applied prior to analysis. The second component was the Osteoporosis Assessment Questionnaire Version 2.0 (OPAQ 2.0) (11), a multiple-choice instrument designed to measure quality of life across physical, emotional, and social functioning domains specific to individuals with osteoporosis. Participants received standardised written instructions prior to questionnaire completion and were encouraged to answer all questions as accurately and completely as possible. Questionnaires with substantial missing data or incomplete responses were excluded from analysis. Moderate-intensity activities were assigned a value of 4 metabolic equivalents (METs) and vigorous-intensity activities a value of 8 METs for the calculation of MET-minutes per week. Standard GPAQ validity checks and truncation procedures were applied to minimise implausible physical activity estimates prior to statistical analysis.

### 2.4 Data analysis

Data were analysed using SPSS Version 29. OPAQ items were scored using the standard scoring algorithm (0–4 scale). Descriptive statistics (frequencies and summary measures) were used to describe participant demographics and physical activity levels (GPAQ). Inferential analyses included chi-square tests, independent samples *t* tests, and correlation analyses. Normality was assessed using the Kolmogorov–Smirnov and Shapiro–Wilk tests. Where assumptions of normality were violated, non-parametric analyses were considered and interpreted accordingly. Descriptive statistics were reported as means and standard deviations. Comparisons of physical activity and quality of life between public- and private-healthcare sector participants were conducted using *t* tests. Statistical significance was set at *p* < 0.05. A *post hoc* power analysis was conducted to evaluate whether the sample size was sufficient to detect significant subgroup differences between public- and private-healthcare participants.

## 3 Results

### 3.1 Demographic information

A total of 209 adults with osteoporosis participated, 89% of whom were female. The mean age was 64.31 ± 11.64 years, with mean height 163.24 ± 8.98 cm, weight 65.45 ± 17.43 kg, and age at diagnosis 52.46 ± 12.48 years (Table 1).

**Table 1:** Demographic data of the sample: age, height, weight and age diagnosed.

Variable	Mean ± SD	Minimum	Maximum
Age (years)	64.31 ± 11.64	35	94
Height (cm)	163.24 ± 8.98	141	200
Weight (kg)	65.45 ± 17.43	31	170
Age diagnosed (years)	52.46 ± 12.48	35	85

### 3.2 Sedentary time

**Figure 1:**
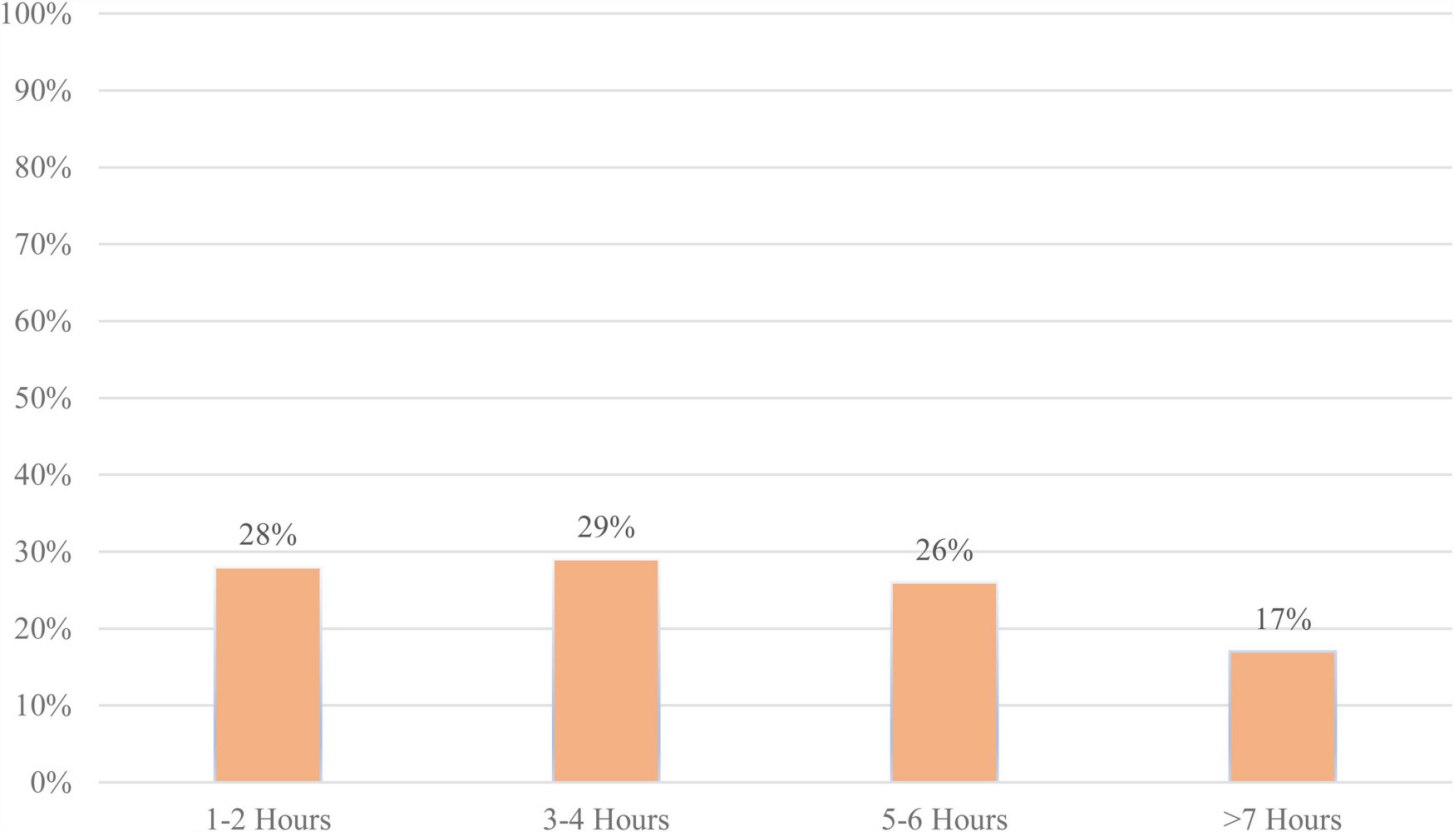
Sedentary time spent seated or reclining on a typical day.

Figure 1 illustrates the extent of sedentary behaviour among participants, measured as the average time spent sitting or reclining per day. Among 204 respondents, 28% reported 1–2 h, 29% 3–4 h, 26% 5–6 h, and 17% more than 7 h of sitting or reclining per day (*p* ≤ 0.05).

### 3.3 Global physical activity questionnaire (GPAQ)

The GPAQ questionnaire measures physical activity in three domains, namely, work, travel and leisure. All domains were analysed at vigorous- and moderate-intensities (Table 2).

**Table 2:** Results of the GPAQ including time spent physically active during work, travel and leisure for both moderate and vigorous intensities.

GPAQ	Participant responses	Participants and percentage (%)	Mean ± SD number of days	Mean ± SD amount of time (minutes)	Significance
Q1–3 Vigorous intensity activity during work	Yes	11 (6%)	3 ± 2	153 ± 122.03	.28
	No	109 (55%)			.09
	Unemployed	78 (39%)			
	Student	0			
Q4–6 Moderate intensity activity during work	Yes	39 (32%)	4 ± 2	124.19 ± 104.29	.06
	No	81 (68%)			.00*
Q7–9 Activity during travel	Yes	78 (40%)	5 ± 2	49.93 ± 44.73	.00*
	No	116 (60%)			.00*
Q10–12 Vigorous intensity activity during leisure	Yes	54 (28%)	4 ± 2	59.67 ± 54.17	.01*
	No	140 (72%)			.00*
Q13–15 Moderate intensity activity during leisure	Yes	80 (41%)	3 ± 2	51.42 ± 49.25	.00*
	No	113 (59%)			.00*

* Indicates significance (*p* ≤ .05).

Vigorous-intensity work activity was reported by 6% of participants (mean 3 ± 2 days/week; 153 ± 122 min/day). Moderate-intensity work activity was reported by 32% (4 ± 2 days/week; 124 ± 104 min/day). Travel-related activity was reported by 40% (5 ± 2 days/week; 50 ± 45 min/day). Vigorous-intensity leisure activity was reported by 28% (4 ± 2 days/week; 60 ± 54 min/day), and moderate-intensity leisure activity by 41% (3 ± 2 days/week; 51 ± 49 min/day). Significant differences were observed for moderate-intensity work activity (*p* < 0.001), travel-related activity (*p* < 0.001), vigorous-intensity leisure activity (*p* = 0.01), and moderate-intensity leisure activity (*p* < 0.001), whereas vigorous-intensity work activity was not statistically significant (*p* = 0.28) (Table 2).

### 3.4 Quality of life (OPAQ)

Quality of life was assessed using the OPAQ, which evaluates physical function, emotional status, and social interaction. Table 3 summarises the results across all domains. OPAQ scores were converted to percentages by dividing the obtained domain score by the maximum possible score for that domain and multiplying by 100.

**Table 3:** Frequency table for osteoporosis physical activity questionnaire (OPAQ).

Domain	Mean ± SD	Minimum	Maximum	Significance
Physical function	66.82 ± 19.95	14	88	0.00*
Physical %	76.05 ± 22.63	16	100	0.00*
Social interaction	24.41 ± 7.31	0	36	0.00*
Social %	55.94 ± 15.75	11	82	0.00*
Emotional status	37.25 ± 13.16	2	64	0.09
Emotional %	58.24 ± 20.49	3	100	0.09
Total	66.17 ± 20.64	11	196	0.00*

* Indicates significance (*p* ≤ 0.05).

In Table 3, the mean scores were: physical function 66.82 ± 19.95 (76.05%), social interaction 24.41 ± 7.31 (55.94%), emotional status 37.25 ± 13.16 (58.24%), and total 66.17 ± 20.64. Statistically significant differences were observed for all domains except emotional status (*p* = 0.09).

## 4 Discussion

Sedentary behaviour and physical inactivity are well-established, independent risk factors for poor skeletal health, contributing to reduced bone mineral density (BMD), increased fracture risk, and diminished quality of life (QoL) across the lifespan (12, 13). The high prevalence of sedentary behaviour observed in this cohort suggests that many individuals with osteoporosis may remain insufficiently active despite the recognised benefits of regular movement and weight-bearing activity for skeletal health. This may reflect barriers such as pain, fear of falling, reduced mobility, limited access to structured exercise opportunities, or inadequate rehabilitation support. Similar trends have been reported internationally, where prolonged sedentary time has been associated with poorer musculoskeletal outcomes and reduced functional independence among older adults and individuals with osteoporosis (14).

The biological mechanisms underpinning these associations have been described in previous experimental and clinical literature. Reduced mechanical loading during prolonged sedentary behaviour has been associated with physiological processes linked to reduced osteoblast activity and increased osteoclastic bone resorption, which may contribute to bone loss. Conversely, physical activity-induced mechanical strain may promote osteocyte signalling, bone formation, and skeletal microarchitectural integrity (13). Importantly, population-based studies suggest that even modest substitutions of sedentary time with light activities, such as walking, may reduce osteoporosis risk, highlighting sedentary behaviour as a potentially modifiable exposure (14). However, the present cross-sectional study cannot establish causal relationships between sedentary behaviour, physical activity, and bone-related physiological outcomes.

Living arrangements emerged as a contextual factor influencing sedentary behaviour. Analyses showed that 67% of nursing home residents spent more than seven hours per day seated, whereas participants receiving assistance reported lower sedentary time. This pattern supports evidence that social support, environmental context, and institutional settings shape physical activity behaviour and functional outcomes in older adults (13, 15, 16). These findings underscore the importance of addressing environmental and social determinants when designing interventions to reduce sedentary behaviour. Recent accelerometer-measured studies have similarly demonstrated associations between objectively measured moderate-to-vigorous physical activity patterns and osteoporosis risk in older adults, reinforcing the importance of incorporating objective physical activity assessment approaches into future osteoporosis research (17).

Physical activity patterns assessed using the Global Physical Activity Questionnaire revealed that moderate-intensity leisure activity was most common, followed by travel-related activity. Nevertheless, only 40% of participants reported walking or cycling for transport, and overall activity levels frequently fell short of World Health Organization recommendations of at least 150 min of moderate-intensity or 75 min of vigorous-intensity physical activity per week (12). This profile mirrors global trends of insufficient physical activity among older adults and reinforces the need for structured and targeted exercise interventions. Recent literature has increasingly emphasised the importance of structured and multicomponent exercise interventions for preserving physical function and quality of life in ageing populations with osteoporosis and related musculoskeletal conditions. Physical activity and exercise have been identified as central components in promoting healthy ageing, functional independence, and improved quality of life among older adults (18). Similarly, emerging evidence from multicomponent and resistance-based exercise interventions suggests that regular physical activity may contribute positively to physical functioning, wellbeing, and skeletal health in older populations at risk of osteoporosis and frailty (19, 20). These findings support the present results and reinforce the importance of incorporating sustainable, context-specific physical activity interventions into osteoporosis management strategies.

High-quality evidence from recent systematic reviews and meta-analyses demonstrates that exercise interventions incorporating resistance training and progressive mechanical loading improve BMD at clinically important sites, including the lumbar spine and femoral neck, while also enhancing QoL in individuals with, or at risk of, osteoporosis (8, 20). These benefits extend beyond observational associations, with randomised trials showing that appropriately prescribed load-bearing and resistance exercises yield measurable physiological and functional improvements and are superior to low-impact activities alone for increasing bone mass and reducing fracture risk (19, 20).

The observed pattern of relatively preserved physical functioning alongside poorer emotional and social quality-of-life domains may suggest that the psychosocial burden of osteoporosis extends beyond functional impairment alone. Fear of falling, reduced confidence, social isolation, and limited participation in meaningful daily activities may contribute substantially to reduced wellbeing among adults with osteoporosis, particularly within settings where rehabilitation and psychosocial support resources are limited (21). These findings align with previous research demonstrating that fractures and mobility limitations have lasting effects on health-related quality of life and daily functioning in osteoporotic populations (12).

Socioeconomic disparities further shaped QoL outcomes, with lower scores observed among public-sector participants. This finding reflects broader international evidence linking socioeconomic deprivation to poorer bone health, higher falls and fracture risk, and reduced QoL in large population cohorts (22). Within the South African context, these disparities may also reflect unequal access to healthcare services, rehabilitation programmes, diagnostic screening, and exercise-based interventions between public and private healthcare sectors. Resource limitations, transportation barriers, and reduced access to biokinetic, physiotherapy, and specialist osteoporosis services may further restrict opportunities for physical activity participation and long-term disease management. Additionally, cultural perceptions surrounding ageing, mobility, and exercise participation among older adults may contribute to sedentary behaviour and reduced engagement in structured physical activity. Emerging data also indicate that osteoporosis independently predicts lower physical components of QoL in older adults, with particularly strong associations observed among women after adjustment for lifestyle and health factors (21). Although osteoporosis research within African populations remains limited, available evidence suggests substantial disparities in osteoporosis diagnosis, healthcare access, rehabilitation opportunities, and physical activity participation across sub-Saharan Africa. Previous South African and African studies have highlighted the influence of socioeconomic inequalities, healthcare accessibility, and contextual environmental factors on bone health outcomes and functional capacity (6, 9). The present findings therefore contribute important local evidence to a relatively underrepresented field and emphasise the need for culturally and contextually appropriate osteoporosis interventions within African healthcare systems.

Taken together, the findings highlight the complex interplay between sedentary behaviour, physical activity, and QoL in individuals with osteoporosis. Prolonged sedentary time and insufficient physical activity remain prevalent, particularly among those with limited social or environmental support, and are recognised as modifiable lifestyle factors with independent adverse effects on bone health and fracture risk (13). Regular engagement in moderate-to-vigorous, weight-bearing physical activity has been associated with reduced vertebral fracture risk and improve bone outcomes in population-based studies (23), while structured exercise interventions consistently improve physical, social, and mental components of QoL, with resistance training demonstrating particularly strong effects (24).

From a clinical perspective, these findings reinforce the need to integrate individualised exercise prescription, strategies to reduce sedentary time, and psychosocial support into comprehensive osteoporosis management. Clinical care should prioritise early lifestyle intervention, ongoing physical activity counselling, and access to structured, supervised exercise programmes. In resource-constrained public-sector settings, low-cost approaches such as walking programmes, body-weight or elastic-band resistance exercises, and community-based group activities represent feasible and effective strategies. Embedding biokineticists, physiotherapists, and other exercise professionals within multidisciplinary care pathways may further enhance skeletal health, reduce fracture risk, and improve patient-centred quality of life across diverse populations.

### 4.1 Limitations

This study has several limitations that should be considered when interpreting the findings. First, the cross-sectional design precludes causal inferences between physical activity, sedentary behaviour, and quality-of-life outcomes. Second, data were self-reported, which may have introduced recall and social desirability bias, particularly in relation to physical activity and sedentary time estimates. Third, participants were recruited from both public and private healthcare sectors, as well as online platforms, which may have introduced selection bias. Individuals accessing healthcare services and online recruitment platforms may differ in socioeconomic status, healthcare access, health literacy, and opportunities for physical activity participation. Consequently, the findings may not be fully generalisable to all individuals with osteoporosis in South Africa, particularly those in underserved or rural settings. In addition, the study was conducted within a single geographic region, which may further limit external validity to other healthcare contexts. Fourth, the inclusion of participants across a broad age range (35–94 years) without age-stratified analyses may have influenced interpretation of physical activity, sedentary behaviour, and quality-of-life outcomes, given the age-dependent nature of osteoporosis, functional decline, and activity behaviour. Fifth, the sample was predominantly female (89%), limiting generalisability to males with osteoporosis. Furthermore, sex differences were not statistically controlled for, limiting the ability to determine whether observed differences in physical activity and quality-of-life outcomes may have been influenced by sex-related factors. Sixth, osteoporosis subtype classification (e.g., primary vs. secondary osteoporosis) was not consistently available, and the inability to distinguish between these forms may have introduced clinical heterogeneity that could have influenced physical activity behaviour and quality-of-life outcomes. Seventh, employment status influenced the applicability of work-related physical activity data, with 39% of participants reporting unemployment. Finally, effect sizes were not calculated for inferential analyses, limiting interpretation of the magnitude and practical significance of observed associations and group differences. Although the sample size was considered adequate for exploratory analyses, subgroup comparisons may have been underpowered for detecting smaller effect sizes.

### 4.2 Recommendations

Based on the results, interventions to reduce sedentary behaviour and promote regular, moderate-to-vigorous physical activity are recommended for adults with osteoporosis, particularly targeting leisure-time and travel-related activity. Healthcare providers should incorporate tailored exercise programmes that emphasise weight-bearing and resistance exercises to improve bone health and physical function. Strategies to enhance social engagement and emotional well-being should also be considered, given the lower scores in these quality-of-life domains. Future research should incorporate longitudinal monitoring protocols, objective physical activity measurement tools such as accelerometry, and intervention studies tailored to the resource constraints commonly experienced within South African public-sector healthcare settings. Additional investigation into gender differences, contextual barriers to exercise participation, and culturally appropriate rehabilitation strategies may further strengthen osteoporosis management within diverse populations.

## 5 Conclusion

This study aimed to determine physical activity levels and quality of life among adults with osteoporosis living in Johannesburg, South Africa. The findings demonstrated that although many participants reported engagement in moderate physical activity, sedentary behaviour remained prevalent. Quality of life was most negatively affected within the emotional and social domains, and lower quality-of-life scores were observed among participants accessing public-sector healthcare services. Increased physical activity participation was generally associated with improved quality-of-life outcomes. These findings contribute important South African data to the limited literature on osteoporosis, physical activity, and quality of life within African contexts. The results support the importance of targeted physical activity promotion, sedentary behaviour reduction strategies, and improved access to rehabilitation and exercise-based interventions in osteoporosis management. These findings should, however, be interpreted in light of the study's cross-sectional design, reliance on self-reported data, and limited geographic representation. Importantly, the present study contributes valuable South African evidence to the limited African literature examining physical activity, sedentary behaviour, and quality-of-life outcomes among adults with osteoporosis.

## Data Availability

The raw data supporting the conclusions of this article will be made available by the authors, without undue reservation.
